# Fall-related activity avoidance in relation to a history of falls or near falls, fear of falling and disease severity in people with Parkinson’s disease

**DOI:** 10.1186/s12883-016-0612-5

**Published:** 2016-06-02

**Authors:** Manzur Kader, Susanne Iwarsson, Per Odin, Maria H. Nilsson

**Affiliations:** Department of Health Sciences, Lund University, PO Box 157, SE-221 00 Lund, Sweden; Department of Clinical Sciences, Lund University, Lund, Sweden; Department of Clinical Sciences, Section for Neurology, Skåne University Hospital, Lund, Sweden; Department of Neurology, Central Hospital, Bremerhaven, Germany; Memory Clinic, Skåne University Hospital, Malmö, Sweden

**Keywords:** Activity avoidance, Falls, Fear of falling, Hoehn and Yahr stages, mSAFFE, Near falls, Parkinson’s disease

## Abstract

**Background:**

There is limited knowledge concerning fall-related activity avoidance in people with Parkinson’s disease (PD); such knowledge would be of importance for the development of more efficient PD-care and rehabilitation. This study aimed to examine how fall-related activity avoidance relates to a history of self-reported falls/near falls and fear of falling (FOF) as well as to disease severity in people with PD.

**Methods:**

Data were collected from 251 (61 % men) participants with PD; their median (min-max) age and PD duration were 70 (45–93) and 8 (1–43) years, respectively. A self-administered postal survey preceded a home visit which included observations, clinical tests and interview-administered questionnaires. Fall-related activity avoidance was assessed using the modified Survey of Activities and Fear of Falling in the Elderly (mSAFFE) as well as by using a dichotomous (Yes/No) question. Further dichotomous questions concerned: the presence of FOF and the history (past 6 months) of falls or near falls, followed by stating the number of incidents. Disease severity was assessed according to the Hoehn and Yahr (HY) stages.

**Results:**

In the total sample (*n* = 251), 41 % of the participants reported fall-related activity avoidance; the median mSAFFE score was 22. In relation to a history of fall, the proportions of participants (*p* < 0.001) that reported fall-related activity avoidance were: non-fallers (30 %), single fallers (50 %) and recurrent fallers, i.e. ≥ 2 falls (57 %). Among those that reported near falls (but no falls), 51 % (26 out of 51) reported fall-related activity avoidance. Of those that reported FOF, 70 % reported fall-related activity avoidance. Fall-related activity avoidance ranged from 24 % in the early PD-stage (HY I) to 74 % in the most severe stages (HY IV-V).

**Conclusions:**

Results indicate that fall-related activity avoidance may be related to a history of self-reported falls/near falls, FOF and disease severity in people with PD. Importantly, fall-related activity avoidance is reported among those that do not fall and already in mild PD-stages (HY I-II). Although further studies are needed, our findings indicate that fall-related activity avoidance needs to be addressed early in order to prevent sedentary behavior and participation restrictions.

## Background

Parkinson’s disease (PD) is a chronic progressive neurodegenerative disease that results in a gradual progression of functional loss and disability due to motor symptoms (i.e. tremor, rigidity, bradykinesia and postural instability) as well as non-motor symptoms (i.e. fatigue, depression, sleep disturbance and cognitive dysfunction) [1, 2]. The severity of PD is most commonly described by using the Hoehn and Yahr (HY) stages [3], which range from stage I (unilateral involvement) to stage V (confinement to bed or wheelchair unless aided). Since PD treatment guidelines [4] often refer to disease severity, it is important to have a thorough understanding of how different problems relate to the HY stages.

People with PD have an increased risk for falling as compared to others of the same age [5]. In studies that used a 6-month recall period, the proportion of fallers ranged from 24 to 67 % [5–7]. Falls have been identified as one of the most disabling features of PD [5, 8]. Studies that involved people with PD have shown that fear of falling (FOF) predicts falls and/or near falls [6] as well as recurrent falls [9]. FOF negatively affects activities of daily living, the level of physical activity [10, 11], health-related quality of life [12] and participation in meaningful activities [13]. It is plausible that the increased risk for falls and FOF could induce fall-related activity avoidance in people with PD.

While there is an increased research attention towards falls and FOF, less is known about fall-related activity avoidance in people with PD [14–16]. The modified Survey of Activities and Fear of Falling in the Elderly (mSAFFE) instrument targets self-rated activity avoidance due to the risk of falling in relation to 17 activities [17]. Previous PD-studies that used the mSAFFE reported that “going out when it is slippery” and “going to a place with crowds” were the two most commonly avoided activities [14–16]. Two previous PD-studies identified that fall-related activity avoidance was associated with a history of previous falls and FOF; the latter assessed by using a dichotomous question [15, 16]. However, none of these studies reported in detail which activities that were avoided among fallers and those reporting FOF. Moreover, to the best of our knowledge, no study has yet investigated how disease severity is related to fall-related activity avoidance in people with PD.

It is also common that people with PD experience near falls, which can be defined as “a fall initiated but arrested by support from a wall, railing, other person, etc.” [18]. Using this definition, the proportions of people with PD that report a history of near falls during a 6-month recall period range from 35 to 45 % [6, 15, 19, 20]. Although previous studies have investigated near falls as a risk factor for future falls [6, 21], they did not report how a history of near falls may relate to fall-related activity avoidance in people with PD.

The aim of this study was to investigate how fall-related activity avoidance relates to a history of self-reported falls/near falls and FOF as well as to disease severity in people with PD; a specific focus addressed which activities that were avoided.

## Methods

The current study used baseline data collected for the project “Home and Health in People Ageing with PD”. Further details regarding the design, inclusion and exclusion criteria, recruitment process, ethical considerations, procedure and data collection were published in a study protocol [22].

### Participants and recruitment

A sample of 653 participants (recruited from three hospitals in Region Skåne, Sweden) met the inclusion criterion of being diagnosed with PD (G20.9) for at least 1 year. Out of these, 216 individuals were excluded according to the following criteria: difficulties in understanding/speaking Swedish (*n* = 10), severe cognitive difficulties (*n* = 91), living outside Skåne (*n* = 58) or other reasons (*n* = 57) (e.g., hallucinations or a recent stroke). The exclusion of participants due to severe cognitive difficulties was done by specialist PD-nurses and screening of medical records. Even if cognitive data was available in many cases, we did not use a specific cut off score regarding global cognitive functioning (e.g., Mini-Mental State Examination or the Montreal Cognitive Assessment) for exclusion. We rather relied on the clinical estimation of the patient’s capacity to give informed consent or take part in the majority of the data collection by the PD-nurse and additional information in the medical records. That is, a potential participant was excluded if not deemed to be able to give an informed consent or partake in the majority of the data collection. Among the remaining 437 individuals who were invited to participate 157 declined, 22 were unreachable, two had their PD diagnosis revised and one was excluded due to extensive missing data. For the present study, another four participants were excluded due to: did not respond to any of the self-administered questionnaires, someone else had in fact responded and severe delays in responding. Accordingly, the study sample consisted of 251 (61 % men) participants; participant’s median (min-max) age was 70 (45–93) years, and the PD duration was 8 (1–43) years. Descriptive information of the total sample is provided in Table 1

**Table 1:** Participant characteristics (*N* = 251)

Variable	Median (q1-q3) unless otherwise stated	Missing value, n
Age (years)	70 (65–77)	-
Sex (men), n (%)	152 (61)	-
Education (elementary/higher secondary/university), n (%)	86 (34)/81 (32)/84 (34)	-
Social support (from partner/other than partner/none), n (%)	156 (62)/92 (37)/3 (1)	-
Living alone (yes), n (%)	66 (26)	-
PD duration (years)	8 (5–13)	-
PD severity (H&Y)	3 (2–3)	-
Freezing (FOGQsa item 3, dichotomized, yes), n (%)^a^	139 (56 %)	3
Motor symptoms (UPDRS III)	30 (22–39)	4
Cognitive function (MoCA)	26 (22–28)	6
Depressive symptoms (GDS-15)	2 (1–4)	5
Fall-related activity avoidance (mSAFFE)	22 (18–31)	11^b^
Fall-related activity avoidance (yes), n (%)	102 (41)	1
Fear of falling (yes), n (%)	121 (48)	1
Falls past 6 months (yes), n (%)	110 (44)	-
Falls past 6 months in relation to H&Y stages (yes), n (%)		
I, *n* = 50	16 (32)	-
II, *n* = 72	29 (40)	-
III, *n* = 67	31 (46)	-
IV + V, *n* = 62	34 (55)	-
Near falls past 6 months (yes), n (%)	141 (57)	3
Near falls past 6 months among non-fallers (yes), n (%)	51 (37)	2

*q1-q3* first-third quartile, *PD* Parkinson’s disease, *H&Y* Hoehn & Yahr (1–5, higher = worse), *FOGQsa* self-administered version of the Freezing of Gait Questionnaire, *UPDRS III* Unified Parkinson’s Disease Rating Scale (motor examination, 0–108, higher = worse), *MoCA* Montreal Cognitive Assessment (0–30, higher = better); *GDS-15* = Geriatric Depression Scale (0–15, higher = worse), *mSAFFE* modified Survey of Activities and Fear of Falling in the Elderly (17–51, higher = more avoidance); ^a^Those who scored ≥1 on item 3 of FOGQsa were classified as having freezing. ^b^Missing value for total scores of mSAFFE

### Data collection and instruments

The data collection included a self-administered postal survey followed by a subsequent home visit that involved interview-administered questions and questionnaires, observations and clinical assessments. The data collection was administered and performed by two trained project administrators (experienced reg. occupational therapists).

#### Fall-related activity avoidance and fear of falling (FOF)

The self-administered modified Survey of Activities and Fear of Falling in the Elderly (mSAFFE) [17] addresses fall-related activity avoidance in relation to 17 activities. Each item (i.e. activity) has three response categories (scored 1–3): never, sometimes, or always avoids. The total mSAFFE score ranges from 17 to 51 (higher = worse). The mSAFFE has been shown to be reliable and valid in people with PD [15, 16]. In addition, two self-administered dichotomous (Yes/No) questions were used. One targeted fall-related activity avoidance: “Do you avoid activities due to a risk of falling?” whereas the other concerned FOF: “Are you afraid of falling?”

#### Falls and near falls

An interview administered dichotomous (Yes/No) question targeted the history of falls during the past 6 months. If the participant answered yes, a subsequent question concerned whether falls had occurred more than once (Yes/No), including providing an estimate of how many times. The European consensus definition of a fall was applied; “an event in which the respondent came to rest on the ground, floor, or lower level” [23]. In this study, a person was defined as a recurrent faller if reporting two or more incidents. A self-administered question (Yes/No) concerned experiences of near falls during the past 6 months, using the following definition: “a fall initiated but arrested by support from a wall, railing, or other person, etc.” [18].

#### Disease severity

Disease severity was assessed (in “on-state”) according to HY [3], which includes five stages: HY I (unilateral involvement only usually with minimal or no functional disability); HY II (bilateral involvement without impairment of balance); HY III (unilateral or bilateral + postural instability); HY IV (severely disabled; still able to walk or stand unassisted); and HY V (confined to bed or wheelchair unless aided).

#### Descriptive variables

Descriptive variables included age, sex, education, type of social support, living alone or not, PD duration, freezing of gait (FOG), motor symptoms, cognitive function, and depressive symptoms. The presence of FOG was assessed by using item 3 (i.e., freezing) of the self-administered version [24] of the Freezing of Gait Questionnaire [25] (FOGQsa); those who scored ≥ 1 were classified as having FOG [15, 20, 26]. Assessments included part III (motor symptoms) of the Unified Parkinson’s Disease Rating Scale (UPDRS III) [27] and the Montreal Cognitive Assessment (MoCA) [28]. Depressive symptoms were assessed with the Geriatric Depression Scale (GDS-15) [29].

### Statistical analyses

Descriptive statistics were computed for all variables. The findings are reported with medians and quartiles to describe ordinal data (deviated from normal distribution, except age) and frequencies and percentages to describe group proportions. HY stage IV (*n* = 56) and stage V (*n* = 6) were merged due to reasons of distribution. Non-parametric tests (the Kruskal-Wallis test and/or Mann-Whitney *U*-test for ordinal variables) or the Chi-Square test for dichotomous or categorical variables were used for sub-group comparisons. Initially, the Kruskal-Wallis or the Chi-Square tests were used for comparisons of more than two sub-groups. If the *p*-value then was statistical significant, subsequent tests (Mann-Whitney tests or additional Chi-Square tests) were corrected for multiple comparisons, using the Bonferroni Correction.

All *p*-values reported are based on two-tailed comparisons where applicable; the alpha level of significance was set at 0.05; *p*-values were presented exactly except when below 0.001. All statistical analyses were computed by using SPSS v. 22 software for Windows (IBM Corporation, Armonk, NY, United States).

## Results

In the total sample (*n* = 251), based on the dichotomous question 41 % reported fall-related activity avoidance; the median mSAFFE score was 22 (ranged from 17 to 50). The highest proportions of participants avoided “Going out when it is slippery” (74 %), “Reaching for something above your head” (50 %), and “Walk a kilometer” (49 %) (see Table 2).

**Table 2:** Activity avoidance (including a ranking order) according to mSAFFE items (*N* = 251)

		Response category, n (%)				Ranking (1–17), most avoided ranked as 1
		Would never avoid	Sometimes avoid	Always avoid	Sometimes + always	
Item					(merged)	
no.	Activity	n (%)	n (%)	n (%)	n (%)	
1	Go to the shops^a^	152(62)	77 (31)	18 (7)	95 (38)	11
2	Clean your house^a^	146 (59)	83 (34)	18 (7)	101 (41)	8
3	Prepare simple meals^b^	187 (76)	54 (22)	5 (2)	59 (24)	14
4	Go to the doctor or dentist^b^	208 (85)	32 (13)	6 (2)	38 (15)	17
5	Take a bath^c^	159 (65)	52 (21)	34 (14)	86 (35)	12
6	Take a shower^a^	194 (79)	45 (18)	8 (3)	53 (21)	15
7	Go for a walk^d^	147 (59)	85 (34)	16 (7)	101 (41)	9
8	**Go out when it is slippery** ^a^	**63 (26)**	**105 (42)**	**79 (32)**	**184 (74)**	**1**
9	Visit a friend or relative^a^	171 (69)	68 (28)	8 (3)	76 (31)	13
10	**Go to a place with crowds** ^d^	**133 (54)**	**85 (34)**	**30 (12)**	**115 (46)**	**4**
11	Go up and down stairs^d^	145 (58)	78 (32)	25 (10)	103 (42)	7
12	Walk around indoors^a^	199 (81)	44 (18)	4 (1)	48 (19)	16
13	**Walk a kilometer** ^b^	**126 (51)**	**67 (27)**	**53 (22)**	**120 (49)**	**3**
14	**Bend down to get something** ^d^	**141 (57)**	**92 (37)**	**15 (6)**	**107 (43)**	**5**
15	Travel by public transport^d^	145 (58)	64 (26)	39 (16)	103 (42)	6
16	Go out to a social event^d^	149 (60)	88 (36)	11 (4)	99 (40)	10
17	**Reach for something above your head** ^d^	**124 (50)**	**92 (37)**	**32 (13)**	**124 (50)**	**2**

*mSAFFE* modified Survey of Activities and Fear of Falling in the Elderly, each item (i.e. activity) has three response categories: never, sometimes or always avoid; Top five avoided activities are marked in bold

^a^ *n* = 4 missing values, ^b^ *n* = 5 missing, ^c^ *n* = 6 missing and ^d^ *n* = 3 missing

### Fall-related activity avoidance in relation to a history of falls, near falls and fear of falling

Overall, the most frequently avoided activity (sometimes or always avoided according to mSAFFE items) was “Going out when it is slippery” (71–95 %). The second most frequently avoided activity was “Go to a place with crowds” for single fallers (52 %) and for those that reported near falls but no falls (61 %). For recurrent fallers and those reporting FOF, the second most frequently avoided activity was “Reach for something above your head” (71 and 75 %, respectively). For further details, see Table 3.

**Table 3:** Activity avoidance (mSAFFE items) in relation to a history falls/near falls, and fear of falling (*N* = 251)

		Falls past 6 months					Near falls^a^ (but no falls) past 6 months			Fear of falling^b^		
Item		No	Single	Rk	Recurrent	Rk	No	Yes	Rk	No	Yes	Rk
no.	Activity^c^ (sometimes + always avoided)	*n* = 141	*n* = 38		*n* = 72		*n* = 88	*n* = 51		*n* = 129	*n* = 121	
1	Go to the shops, n (%)	36 (26)	15 (40)	10	44 (61)	**5**	14 (17)	22 (43)	11	18 (14)	77 (65)	6
2	Clean your house, n (%)	42 (31)	15 (40)	9	44 (61)	6	17 (20)	25 (49)	8	28 (22)	73 (62)	11
3	Prepare simple meals, n (%)	19 (14)	9 (24)	14	31 (44)	14	7 (8)	12 (24)	15	12 (9)	47 (40)	14
4	Go to the doctor or dentist, n (%)	17 (12)	5 (13)	17	16 (23)	17	6 (7)	11 (22)	16	7 (5)	31 (27)	17
5	Take a bath, n (%)	39 (29)	13 (34)	12	34 (48)	12	16 (19)	23 (46)	10	23 (18)	63 (54)	12
6	Take a shower, n (%)	27 (20)	5 (13)	16	21 (29)	16	10 (12)	17 (33)	14	10 (8)	43 (36)	15
7	Go for a walk, n (%)	44 (32)	16 (42)	7	41 (57)	9	17 (20)	27 (53)	6	27 (21)	74 (62)	9
8	Go out when it is slippery, n (%)	96 (70)	27 (71)	**1**	61 (86)	**1**	51 (59)	45 (88)	**1**	72 (56)	112 (95)	**1**
9	Visit a friend or relative, n (%)	32 (23)	12 (32)	13	32 (44)	13	13 (15)	19 (38)	13	17 (13)	59 (50)	13
10	Go to a place with crowds, n (%)	52 (38)	20 (52)	**2**	43 (60)	7	21 (24)	31 (61)	**2**	33 (26)	82 (69)	**4**
11	Go up and down stairs, n (%)	50 (36)	15 (39)	11	38 (53)	10	25 (29)	25 (49)	7	26 (20)	77 (65)	7
12	Walk around indoors, n (%)	17 (12)	9 (24)	15	22 (31)	15	8 (9)	9 (18)	17	12 (9)	36 (31)	16
13	Walk a kilometer, n (%)	56 (41)	19 (50)	**4**	45 (63)	**4**	26 (30)	30 (59)	**3**	35 (27)	85 (72)	**3**
14	Bend down to get something, n (%)	48 (35)	16 (42)	8	43 (60)	8	24 (28)	24 (47)	9	31 (24)	76 (64)	8
15	Travel by public transport, n (%)	37 (27)	20 (52)	**3**	46 (64)	**3**	16 (18)	21 (41)	12	24 (19)	79 (66)	**5**
16	Go out to a social event, n (%)	45 (33)	19 (50)	**5**	35 (49)	11	18 (21)	27 (53)	**5**	25 (19)	74 (62)	10
17	Reach for something above your head, n (%)	56 (41)	17 (45)	6	51 (71)	**2**	27 (31)	29 (57)	**4**	35 (27)	89 (75)	**2**

*mSAFFE* modified Survey of Activities and Fear of Falling in the Elderly, each item (i.e. activity) has three response categories: never, sometimes or always avoid; the response categories sometimes and always are merged; Rk = Ranking order (1–17; 1 denotes the most avoided activity). Top five avoided activities are marked in bold

^a^ *n* = 2 missing values, ^b^ *n* = 1 missing and ^c^ *n* = 3–6 missing (For further details regarding missing data see footnote in Table 2

The extent of fall-related activity avoidance differed significantly (*p* < 0.001) among those reporting no falls, a single fall or recurrent falls (see Table 4); the median mSAFFE score was 20, 25 and 28, respectively. Subsequent Mann-Whitney U-tests (Bonferroni correction criterion *p* < 0.016) showed that fall-related activity avoidance was significantly higher in recurrent fallers as compared to the other two sub-groups. There was no statistical significant (*p* = 0.295) difference between those that reported no falls and those that reported a single fall. Moreover, the proportions of participants that reported fall-related activity avoidance differed significantly (*p* < 0.001) among those that reported no falls (30 %), a single fall (50 %) or recurrent falls (57 %). After Bonferroni correction (*p* < 0.016), the proportions between those that reported no falls versus recurrent falls were significantly (*p* < 0.001) different (see Table 4).

**Table 4:** Fall-related activity avoidance in relation to a history falls/near falls^a^, fear of falling^b^ and disease severity (*N* = 251)

Variable	Fall-related activity avoidance			
	mSAFFE^c^		Dichotomous question (yes)	
	median (q1-q3)	*p*-value	n (%)	*p*-value
History of fall		<0.001^*^		<0.001*
No fall, *n* = 141	20 (18–28)^d^		42 (30)^b^	
Single fall, *n* = 38	25 (17–31)		19 (50)	
Recurrent falls (>1), *n* = 72	28 (22–35)^e^		41 (57)	
History of near fall		<0.001		<0.001
No near falls or falls, *n* = 88	19 (17–22)^f^		15 (17)	
Near falls, but no falls, *n* = 51	25 (19–33)^a^		26 (51)	
Fear of falling		<0.001		<0.001
No, *n* = 129	19 (17–22)^f^		17 (13)	
Yes, *n* = 121	30 (23–35)^d^		85 (70)	
Disease severity, HY stages		<0.001^**^		<0.001**
I, *n* = 50	19 (17–25)		12 (24)	
II, *n* = 72	19 (17–24)^b^		14 (19)	
III, *n* = 67	23 (19–31)^f^		31 (46)	
IV+ V, *n* = 62	32 (26–39)^d^		45 (74)^b^	

*q1-q3* first-third quartile, *mSAFFE* modified Survey of Activities and Fear of Falling in the Elderly (17–51, higher = more avoidance), *HY* Hoehn and Yahr (1–5, higher = worse)

*All subsequent unpaired comparisons showed a statistical significant difference (Bonferroni correction criterion of *P* < 0.016) except between no fall-single fall for mSAFFE, and except between no fall-single fall and single fall-recurrent fall for dichotomous question

**All subsequent unpaired comparisons showed a statistical significant difference (Bonferroni correction criterion of *P* < 0.0083) except between HY I-II for mSAFFE, and except between HY I-II and I-III for the dichotomous question

^a^ *n* = 2 missing values, ^b^ *n* = 1 missing, ^c^ *n* = 11 missing, ^d^ *n* = 7 missing, ^e^ *n* = 4 missing and ^f^ *n* = 3 missing

Those that reported a history of near falls (but no falls) reported significantly (*p* < 0.001) more fall-related activity avoidance than those without such incidents; the median (q1-q3) mSAFFE score was 25 (19–33) versus 19 (17–22). The corresponding proportions of participants that reported fall-related activity avoidance were 51 versus 17 % (*p* < 0.001), (see Table 4).

Those with FOF reported significantly (*p* <0.001) more fall-related activity avoidance than those without (median mSAFFE score was 30 versus 19); the proportions of participants that reported fall-related activity avoidance were 70 versus 13 % (*p* < 0.001) (see Table 4).

### Fall-related activity avoidance in relation to disease severity

The extent of fall-related activity avoidance differed significantly (*p* < 0.001) in relation to disease severity (see Table 4); the median (q1-q3) mSAFFE score ranged from 19 (17–25) in HY I to 32 (26–39) in HY stages IV-V. Subsequent Mann-Whitney U-tests (Bonferroni correction criterion of *p* < 0.0083) showed significant differences for all comparisons except between HY stages I and II. The proportion of participants that reported fall-related activity avoidance was significantly (*p* < 0.001) higher in the more severe disease stages; it mounted to 74 % in the most severe group. The subsequent comparisons were statistically significant (Bonferroni criterion of *p* < 0.0083), except between stages HY I and II and I and III.

## Discussion

Our study suggests that people with PD with a history of self-reported falls or near falls and FOF report significantly more fall-related activity avoidance than those without. Moreover, those that do not fall also report fall-related activity avoidance. People with PD seem to avoid activities that they presumably consider as being risky (e.g., going out when it is slippery), which can be a sound strategy. However, it is also common to avoid activities such as walking 1 km or activities that involve situations with large numbers of people (e.g., crowds and public transport), indicating that people with PD may be at risk for restricted participation in society. Although fall-related activity avoidance seems to increase with an increased severity of PD, it is noteworthy that it is reported in HY stages I and II. Accordingly, this suggests that fall-related activity avoidance needs to be addressed early in order to prevent sedentary behavior and participation restrictions.

As to the level of detail regarding activity avoidance as assessed by mSAFFE, our findings extend those reported by Rahman et al. [14]. That is, the present results add more detailed knowledge regarding whether the participants sometimes or always avoid the activities and identify which activities that are avoided if reporting falls, near falls (but no fall) and FOF. To the best of our knowledge, this is the first PD-study that reports such details of fall-related activity as well as how it relates to PD severity. In addition, as compared to previous PD-studies that used the mSAFFE (n ranged from 20 to 130) [14–16, 30], our study included the largest sample size.

In agreement with previous PD-studies [14–16], the most frequently avoided activity due to the risk of falling was “Going out when it is slippery”. In our total sample the second most frequently avoided activity was “Reaching for something above your head” whereas”Go to a place with crowds” was noted in other PD studies [14–16]. In our total sample “Go to a place with crowds” was ranked fourth, while it was ranked as the second most commonly avoided activity among single fallers, as well as among those that reported near falls but no fall. Such discrepancies among studies might be explained by methodological differences regarding inclusion- and exclusion criteria, recruitment procedures, sample characteristics and analysis approaches.

It needs to be noted that the ranking order between some items mirror only small differences and should therefore not be given unmotivated attention. For example, after merging two response categories (sometimes, always avoided), in the total sample there is only 1 % difference between the activity ranked as 2 (“Reach for something above your head”) and the activity ranked as 3 (“Walk a kilometer”). The important message here is that more than one fifth of our participants reported that they always avoid walking a kilometer due to the risk of falling, highlighting the importance of addressing fall-related activity avoidance in order to promote physical activity.

In the present study, fallers reported more fall-related activity avoidance than non-fallers. This corroborates the findings of previous studies using samples that targeted people with PD [14, 16] as well as older community-living people in general [31]. Fall-related activity avoidance was related to FOF which is in accordance with a previous PD-study that used a dichotomous FOF-question [16]. Importantly, a novel and interesting finding is that fall-related activity avoidance is more prevalent and pronounced among those that report near falls (but no falls) as compared to those without any near falls or fall incidents. In fact, our results show that participants without a history of falls report fall-related activity avoidance. This suggests that researchers and clinicians should pose questions not only about falls, but also about near falls, as well as address whether people with PD report fall-related activity avoidance, irrespective of whether they report fall incidences. Moreover, our findings indicate that fall-related activity avoidance is related to PD severity. An important and novel finding is that people with PD report fall-related activity avoidance already in HY stages I and II, which suggests that fall-related activity avoidance needs to be addressed early.

Based on the cross-sectional study design used in this study we were not able to determine any causal directions of the relationships investigated. With the ambition to follow up our sample longitudinally [22], we will later on be in a position allowing for further studies with the potential to explore these intriguing dynamics. We acknowledge that other variables than those investigated in the present study may also be of interest. For example, Rahman et al. considered four potential explanatory factors and found that anxiety independently contributed to fall-related activity avoidance [14]. Future longitudinal studies that use a multivariate analysis should preferably consider a broad variety of factors (e.g. motor and non-motor symptoms, environmental aspects and personal factors) that may contribute to fall-related activity avoidance in people with PD. Turning to another study limitation related to the questions used in this type of studies, self-reported data are subject to recall bias and might be influenced by either an over- or under-estimation by the individual [32].

## Conclusions

Fall-related activity avoidance seems to be related to a history of self-reported falls/near falls, FOF and disease severity in people with PD. Importantly, fall-related activity avoidance is reported also among those that do not fall and already in the early phases of PD. Our findings suggest that fall-related activity avoidance should be addressed early and irrespective of whether people with PD report falls in order to prevent sedentary behavior and participation restrictions. Further studies are needed that use multivariate analysis and have a longitudinal design.
